# Factors associated with successful vaginal birth after cesarean section among mothers who gave birth in Ambo town, Oromia, Central Ethiopia, a case-control study

**DOI:** 10.4314/ahs.v22i4.41

**Published:** 2022-12

**Authors:** Negash Kassa Mamo, Desta Melese Siyoum

**Affiliations:** 1 School of Nursing and Midwifery, Asrat Woldeyes Health Science Campus, Debre Berhan University, Debre Berhan, Ethiopia; 2 Department of Midwifery, College of Medicine and Health Sciences, Hawassa University, Hawassa, Ethiopia

**Keywords:** Previous cesarean section, vaginal birth after cesareans, Ambo, Ethiopia

## Abstract

**Background:**

While Trial of labour after previous cesarean section for women with no contraindication for vaginal delivery is an important strategy to reduce short- and long-term morbidity related with repeated cesarean section, the rate of cesarean section and repeated cesarean section with its complication are increasing.

**Objective:**

The objective of the study was to assess factors associated with successful vaginal birth among women with previous cesarean delivery in public hospitals of Ambo town in 2019.

**Methods:**

A case-control study was conducted in Ambo public Hospitals in 2019. A total of 243 clients were included, of which 81 clients were cases and 162 clients were controls with controls to cases ratio of 2. A Lottery method was applied to select the controls and cases were selected consecutively. Bivariate, followed by multivariate analyses, were conducted with 95% CI and p-value <0.05 to identify factors associated with successful vaginal birth after cesarean delivery.

**Result:**

Parity three and four(AOR = 3.45, 95%CI(1.16, 10.229), labour monitoring with partograph (AOR= 4.77, 95%CI: 1.65,13.88), prior vaginal birth after caesareans (AOR = 5.68, 95%CI:1.44,22.46), occiput-posterior position (AOR = 0.109, 95%CI: (0.02, 0.49), duration of labour after admission less than 8 hours (AOR = 4.57, 95%CI: 1.92,10.85) and artificial rupture of membrane (AOR = 5.02 95%CI: 1.67,15.06) were factors significantly associated with successful vaginal birth after previous cesarean section.

**Conclusion:**

The study shows that parity, duration of labour, previous history of successful vaginal birth after cesarean section, artificial rupture of membrane, and partograph monitoring of labour were associated with successful vaginal birth after previous cesarean section. All Women with a history of cesarean section should be counselled and encouraged to undergo a trial of labour as long as it is not contraindicated.

## Introduction

Trial of labour after cesarean is a planned attempt to deliver vaginally by a woman who had a previous cesarean section, regardless of the outcome. Previously it was thought that if a woman had one cesarean delivery, all future pregnancies should be delivered in cesarean delivery. Currently, it is known that many women can undergo a trial of labour after cesarean delivery (TOLAC) 1–3. Cesarean section is a major surgical procedure undertaken today in the world and currently, 18.6% of all births occur by cesarean section (CS), ranging from 6% to 27.2% in the least and most developed regions, respectively 4.

Despite the WHO's recommendation of cesarean delivery rate of 10% - 15%, currently one in three (33%) women underwent a caesarean section, and both under and overuse of cesarean section equally leads to maternal and perinatal mortality and morbidity 3,5. The continued increase in primary cesarean deliveries for dystocia, failed induction, malpresentation, diabetes mellitus, multiple gestation, the increased practice of cesarean delivery on maternal request; and limited use of a trial of labour after cesarean (TOLAC) delivery due to both safety and medico-legal concerns were among the reasons for the ever-increasing rate of cesarean section worldwide 5–7.

Labour after cesarean delivery is safe and appropriate for most women with a history of one or two prior cesarean births 1–3. Different studies have reported that the general success rate of vaginal birth after birth (VBAC) lies in the range of 50–80 %. Age <30 years, body, prior vaginal delivery, previous VBAC, and absence of a recurrent indication for cesarean section, advanced cervical opening, effacement, gravidity, parity, were associated with successful VBAC and recurrent cause for previous cesarean, short inter-pregnancy interval, and in vitro fertilization pregnancies were factors which negatively affects the success rate of VBAC 8–12

In recent years the rate of cesarean section has been increasing in Ethiopia. According to the national demographic health survey of Ethiopia, the overall percentage of deliveries attended by the caesarean section was 2% 13. A study in eastern Ethiopia the prevalence of cesarean section was reported to be 34.3% 14. Studies conducted in Bahirdar and Addis Ababa have reported that the prevalence of Cesarean section was 25.4 % and 19.2% respectively 15,16. A retrospective study conducted in Jimma found that the prevalence of cesarean delivery was 28.1% and age, address of mothers, antenatal care (ANC) follows up, the number of fetuses, and history of previous CS were factors associated with cesarean section 17. In Ethiopia, studies have been conducted with an emphasis on cesarean section, however, vaginal birth after previous cesarean section and associated factors were not adequately studied, therefore this study is aimed to assess factors associated with successful vaginal birth after cesarean section.

## Methods

### Study Design and Period

Institution based unmatched case control study was conducted from June to December, 2019.

### Study Area

The study was conducted in public Hospitals of Ambo town. Ambo town is the capital of West shoa zone located at 114 km West of Addis Ababa. There are two public hospitals in the town Ambo University Referral Hospital and Ambo general Hospital. The two Hospitals offer a full range of comprehensive emergency obstetric care services such as operative delivery and serve for around 3.5 million residents of West Shoa zone, South West Shoa zone and neighbouring East Wollega zone.

### Study Population

All mothers who had one previous cesarean section and allowed for trial of labour during the study period was the study population of the study. Cases were mothers with previous scar, allowed VBAC, started labour spontaneously, and delivered vaginally. Controls were mothers who delivered by cesarean section after trial of labour.

### Eligibility Criteria

#### Inclusion Criteria

Mothers who had one previous cesarean section and allowed for trial of labour were included.

#### Exclusion Criteria

All mothers who were critically ill and unable to respond questions, referred from other health institution with complication or incomplete documentation were excluded.

### Sample Size Determination and Sampling Producers

The sample size was determined using the sample size calculator of EPI version 7.1 for double population. The sample size was calculated with a 95% confidence interval and detecting the power of 80% with control to cases ratio of 2 and odds ratio of 2.54 and proportion of case among the exposed group of 20.4%. The maximum sample size 221 and considering a 10% non- response rate the final sample size was 243 of which 81 were cases and 162 were controls. Cases were selected consecutively with proportional allocation to each hospital. If there was more than one case present on the same day lottery method was applied to select cases. Controls were selected by lottery methods.

### Data Collection Tools and Procedures

The questionnaire consists of different parts that were developed from different previous literature and modified according to the local context. A structured questionnaire was used to collect the data through a face-to-face interview for the socio-demographic, presence of co-morbid medical illness in the past and present obstetric variables in the postnatal ward before the patient was discharged. Some of the current obstetrics information was obtained from the client's chart. The data collection tool was translated from English to Afaan Oromo for better understanding, then retranslated back to English by professional translators to check for consistency. Data were collected by four trained BSc midwives (who are fluent in local dialect) and two MSc midwives were assigned for supervision. The collected data were checked for completeness and consistency by the data collection supervisor on a daily basis.

### Data quality assurance and data analysis

Training was provided for two days about the objective of the study, the definition of terms in the questionnaire, issues of confidentiality and privacy, and data collection method. The collected data were checked for completeness and consistency by the supervisors and principal investigator. The data was checked for its completeness, coded, cleaned, and entered to Epi info version 7.1.2.0 then transferred to SPSS-version 25 for analysis. Appropriate descriptive analysis (frequency, mean, standard deviation, and charts) were used to present the socio-demographic variables. A Chi-square test was used to identify significant characteristics difference among cases and controls. Factors associated with successful VBAC were analysed using a logistic regression model after checking for the goodness of fit test (Hosmer and Lemeshow test). All variables with a p-value <0.05 in the bivariate analysis were included in multivariate analysis to adjust for potential confounders associated with successful vaginal birth after previous cesarean section. Odds ratios and 95% confidence interval (CI) were computed. Variables with a p-value of <0.05 were considered factors significantly associated with successful vaginal birth after previous cesarean section in multivariate analysis.

### Study variables

#### Dependent Variables

Successful vaginal birth after previous cesarean section

#### Independent Variables

Socio-demographic factors, age, education, income, occupation

Past obstetric variables, parity, gestational age

#### Current obstetric variables

membrane status at admission cervical dilation at admission duration of labour presence of meconium, position gestational age, birth weight, station, ANC follow up

### Operational definition

#### Trial of labour after cesareans

Attempt to deliver vaginally by a woman who had previous cesarean delivery, irrespective of the outcome.

#### Successful vaginal birth after cesarean section

Vaginal birth following trial of labour in a woman who had a prior caesarean section delivery.

## Result

### Socio-demographic characteristics

A total of 239 women have participated in the study with a response rate of 98.35%. The mean (SD) age of the participants was 27.6(±5) for the cases and 28.1(±4.8), for controls. Among the respondents, 51(63.8%) of cases and 101(63.5%) of controls were in the age between 25 and 35 years of age. The respondent's age ranges from 18 to 42 years. Around half 120 (50.2%) of the mothers were housewives and 35(14.6%) do not have formal education. Monthly income had a significant difference among cases and controls. Age, educational status, occupation, and residence were comparable characteristics in cases and controls (Table 1).

**Table 1:** Socio-demographic characteristics of mothers who gave birth after previous cesarean section in Ambo public Hospitals in 2019

Variable	Case (n, n %)	Control (n, n %)	Total	χ2 P-value
Age of the mother <25	18(22.5)	46(28.9)	64(26.8)	0.188
25–35	51(63.8)	101(63.5)	156(63.6)	
>35	11(13.7)	12(7.5)	23(9.6)	
Maternal education No formal education	12(15)	23(14.5)	35(14.6)	0.567
Primary	24(30)	61(38.4)	85(35.6)	
Secondary	20(25)	38(23.9)	58(24.3)	
college and above	24(30)	37(23.9)	61(25.5)	
Ethnicity Oromo	39(48.7)	75(47.2%)	114(47.7)	
Amhara	18(22.5)	38(23.9)	56(23.4)	
Gurage	12(15)	28(17.6%)	40(16.7)	
Wolayta	8(10)	11(6.9%)	19(7.9)	
Others*	3(3.3)	7(4.4)	10(4.2)	
Mothers' occupation House wife	39(48.8)	81(50.9)	120(50.2)	0.469
Government employee	22(27.5)	33(20.8)	55(23)	
Private work	19(23.8)	45(28.3)	64(26.8)	
Monthly income <1000	6(7.5)	26(16.4)	32(13.4)	0.0001
1001–2500	10(12.5)	54(34)	64(26.8)	
2501–3999	27(33.8)	40(25.2)	67(28)	
>4000	37(46.7)	39(24.5)	76(31)	
Residence Urban	58(73.8)	108(67.9)	167(69.9)	0.219
Rural	21(26.2)	51(32.1)	72(30.1)	

Others* includes, Tigre, Kemebata

### Past obstetric characteristics

**Figure 1:**
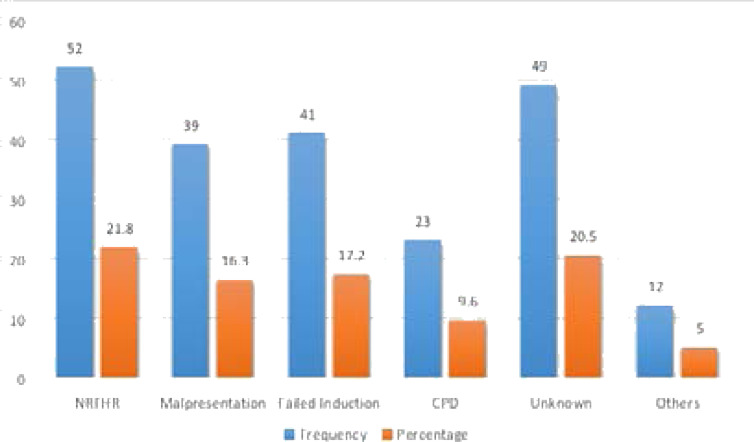
Indications for previous cesarean section among mothers who offered trial of labour in Ambo public Hospitals in 2019.

Fifty-two (67.2%) of cases had three and more birth history and among controls, more than half (59.7%) of mothers had a history of only two births. Parity and history of prior successful VBAC had a significant difference between the cases and controls, other variables were comparable in both the cases and control groups as shown (Table 2). Among the respondents, 51(21.8%) women reported that the indication for their previous cesarean section was NRFHR and 49 (20.8%) women couldn't remember what the indication was (Figure 1).

**Table 2:** Past obstetric characteristics of mothers who gave birth after previous cesarean section in Ambo public Hospitals, 2019

Variable	Case (n, %)	Control (n, %)	Total	χ2 p value
Parity II	28(35)	95(59.7)	123(51.5)	0.001
III –IV	28(35)	36(22.6)	64(26.8)	
Above IV	24(30)	28(17.6)	52(21.8)	
Gestational age in weeks Preterm	9(11.2)	9(11.2)	20(8.4)	0.288
Term	48(60)	48(60)	119(49.8)	
Post term	8(10)	8(10)	17(7.1)	
Unknown	15(8.8)	15(8.8)	83(34.7)	
History of still birth Yes	7(8.8)	24(15.1)	31(13)	0.119
No	73(91.2)	135(84.9)	208(87)	
Vaginal birth before c/s Yes	36(45)	59(37.1)	95(39.7)	0.15
No	44(55)	100(62.9)	144(60.3)	
Vaginal birth after c/s Yes	47(58.8)	14(8.8)	61(19.7)	0.0001
No	33(58.8)	145(91.2)	192(80.3)	

### Current obstetrics characteristics

**Figure 2:**
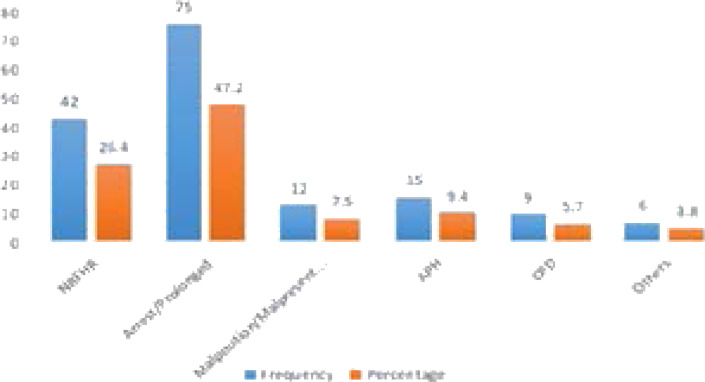
Indications of cesarean sections among mothers who offered trail of labor in Ambo public Hospitals in 2019.

All mothers included in the cases as well as in the controls had antenatal care (ANC) follow up at least once in this pregnancy. Among mothers who have ANC visit about 68.8% of the cases and 53.2% of the controls had four and above visit. Around 81(50.9%) of controls and 33(41.2%) of cases came to the hospitals at the gestational age of less than 40 weeks. Fifty-four (67.5%) of cases an68(42.8%) of controls were admitted in the active phase of labour. Among cases and controls, there was no significant difference in place of ANC, estimated fetal weight, neonatal weight, and neonatal outcomes (Table 3). Among controls, the most common indication for CS is prolonged labour 65(40.9%) followed by Non-reassuring fetal heart rate 46 (28.9%)(Figure 2).

**Table 3:** Current obstetric characteristics of mothers who gave birth after previous cesarean section in Ambo public Hospitals, 2019

Variable	Case (n, %)	Control (n. %)	Total	χ2 p value
Do you have ANC follow up? Yes	80(100)	159(100)	239(100)	
No	0	0	0	
Number of ANC visit <4 times	25(31.2)	76(47.8)	101(42.3)	0.05
4 times	40(50)	55(35.3)	95(39.7)	
>4 times	15(18.8)	28(17.9)	43(18)	
Gestational age in weeks <40	33(41.2)	81(50.9)	114(47.7)	0.209
40	25(31.2)	38(23.9)	63(26.4)	
>40	10(12.5)	26(16.4)	36(15.1)	
Unknown	12(15)	14(8.8)	26(10.9)	
Cervical dilatation at admission <4	57(71.2)	143(89.9)	200(83.7)	0.0001
≥4	23(28.8)	16(10.1)	39(16.3)	
Position of presenting part Occiput Anterior	53(66.2)	45(28.3)	98(41)	0.0001
Occiput Posterior/Transverse	8(10)	27(17)	35(14.6)	
Unknown	19(23.8)	87(54.7)	106(44.4)	
ROM at admission Yes	16(20)	53(33.3%)	69(28.9)	0.035
No	64(80)	106(66.7%)	170(71.7%)	
Duration of rupture <12 hours	14(87.5)	51(96.2)	65(94.2)	
≥12 hours	2(12.5)	2(3.8)	4(5.8)	
Presence of meconium Yes	1(6.2)	7(13.2)	8(11.6)	
No	15(93.8)	46(86.8)	61(88.4)	
Labor followed by partograph Yes	72(90)	75(47.2)	147(61.5)	0.0001
No	8(10)	84(52.8)	92(38.5)	
Duration of labor after admission <8 hours	≥8(35)	114(71.4)	142(59.4)	0.0001
≥8 hours	52(65)	45(28.6)	97(40.6)	
Was ARM done? Yes	34(42.5)	18(11.3)	52(21.8)	0.0001
No	46(57.5)	141(88.7)	187(78.2)	
Weight of neonate <2500	3(3.8)	7(4.4)	10(4.2)	
2500–4000	76(95)	148(93.1)	222(92.9)	
>4000	1(1.2)	4(2.5)	7(2.9)	

### Factors associated with successful VBAC

Monthly income, parity, prior VBAC, number of ANC visit, cervical dilatation, the position of the presenting part, status of the membrane at admission, station, partograph utilization, duration of labour, and ARM were significantly associated with successful vaginal birth after previous cesarean section in bivariate analysis. To control the potential confounding effect, those variables having a p-value less than 0.5 in the bivariate analysis were entered into multiple logistic regression with enter method. Parity three and four, prior successful vaginal birth after cesarean section, partograph followed labour, duration of labour after admission and artificial rupture of the membrane during labour were significantly associated with successful VBAC after adjusting for other variables in multivariate analysis (Table 4).

**Table 4:** Logistic regression analysis of factors associated with successful vaginal birth after previous cesarean section among mothers who gave birth in Ambo public Hospitals, 2019

Variable	Case	Control	COR (95% CI)	AOR (95%CI)
Age <25	18	46	0.43(0.17,1.13)	0.96(0.35, 2.65)
25–35	51	101	0.55(0.20,1.14)	0.53(0.10, 2.79)
>35	11	12	1	
Monthly income <1000	6	26	0.24(0.90,0.658)	
1001–2500	10	54	0.20(0.087, 0.439)	0.21(0.04, 1.09)
2501–3999	27	40	0.77(0.336, 1.382	0.389(0.12, 1.23)
>4000	27	39	1	
Parity II	28	95	1	
III-IV	28	36	2.64(1.38,5.05)	3.45(1.16, 10.23) *
Above IV	24	26	2.91(1.46,5.79)	5.24(0.97, 28.33)
Prior VBAC Yes	33	14	7.27(3.59,14.74)	5.68(1.44, 22.46) *
No	47	145	1	
ANC Visit <4 times	25	76	1	
4 times	40	55	2.211(1.15, 3.91)	1.459(0.58, 3.67)
>4 times	15	28	1.628(0.07, 3.39)	
Cervical dilatation at admission <6cm	57	143	1	
≥6cm	23	16	3.606(1.78, 7.32)	1.406(0.43, 4.65)
Membrane status Ruptured	16	53	2(1.06,3.79)	2.053(0.66, 6.37)
Intact	64	106	1	
Position of the presenting part Occiput Anterior	53	45	1	
Occiput Posterior	8	27	0.252(0.10, 0.61)	0.109(0.02, 0.49) *
Unknown	19	87	0.185(0.10, 0.35)	0.24(0.089, 0.67) *
Station < 0	41	107	1	
≥ 0	32	38	2.198(1.22,3.97)	1.64(0.63, 4.32)
Unknown	7	14	1.305(0.49,3.46)	
labour followed by partograph Yes	72	75	10.08(4.56,22.33)	4.78(1.65, 13.88) *
No	8	84	1	
Duration of labour <8hrs	28	114	4.75(2.71,8.36)	4.56(1.92, 10.85) *
≥8hrs	52	45	1	
Was ARM done? Yes	34	18	5.79(2.99,11.22)	5.02(1.67, 15.07) *
No	46	141	1	

* P-value less than 0.05

## Discussion

The purpose of the study was to identify factors associated with successful vaginal birth after a previous cesarean section among mothers who were eligible for a trial of labour after previous cesarean delivery in Ambo town hospitals. In this study, parity it was found to be associated with successful vaginal birth after previous cesarean section. Women who had three and four deliveries were more than threefold more likely to give successful vaginal birth than women who had two deliveries. Consistently a Nigerian study reported that parity was a significant predictive factor for mode of delivery; the study sated that women with low parity were more likely to have caesarean delivery than those with high parity 18. Similarly, other studies and guidelines reported that parity was found to be an important factor associated with the success of VBAC and the success rate of VBAC increases with higher parity 5,11,12.

Previous successful VBAC was a significant factor for a successful vaginal birth after previous cesarean section. Mothers who had previous successful vaginal birth after cesarean section were five times more likely to give successful vaginal birth than mothers who had not had previous successful vaginal birth after cesarean section. In line with this study, a case-control study conducted in Addis Ababa reported that previous history of successful vaginal birth after a previous cesarean section was determinant of successful vaginal birth after previous cesarean section 19. The ACOG reported that the success rate of VBAC among patients with prior vaginal birth after cesarean delivery is 93% 20. According to the RCOG report previous vaginal delivery, particularly previous successful vaginal birth after previous cesarean section is the single best predictor for a successful vaginal birth after a previous cesarean section and is associated with a planned vaginal birth after previous cesarean section success rate of 85–90 % 10.

Artificial rupture of membrane (ARM) was significantly associated with successful vaginal birth after previous cesarean section. Women with whom their labour was augmented with artificial rupture of the membrane were 5 times more likely to have successful VBAC than their counterparts. Correspondingly a systematic review has reported that early amniotomy was associated with the reduction of cesarean delivery rate compared with routine care 21. The WHO recommended that the artificial rupture of the membrane for labouring mother is one method of augmentation of labour by stimulating the uterus to increase the frequency, duration, and intensity of contractions after the onset of spontaneous labour and reduces the likelihood of delivering by caesarean section 3. This might be related to the effect of labour augmentation secondary to amniotomy. Amniotomy is known to releases prostaglandins from the amniotic fluid and the prostaglandins may increase the frequency and intensity of contractions which results in a shortening of the duration of labour.

The position of the presenting part was also another factor associated with successful vaginal birth after cesarean section. Being occiput posterior position was 89.1% less likely to have a successful VBAC compared with being occiput anterior position. A study in Addis Ababa reports that having an occiput-posterior/transvers position was associated with low success for a vaginal birth after previous cesarean section 19.

In this study duration of labour was found to be a factor significantly associated with successful vaginal birth after previous cesarean section. Mothers whose duration of labour was less than 8 hours after admission was more than four times more likely to give vaginal delivery than mothers whose duration of labour was greater than 8 hours. A study in Tunisia reported that duration of labour greater than 8 hours was associated with poor prognostic factors for a successful vaginal birth after previous cesarean section 22. Another study congruently reported that early admission to labour was associated with a significantly increased risk of delivery by cesarean section 23.

In the current study, partograph utilization was associated with successful vaginal birth after previous cesarean section. Compared to women whose labour was not followed by partograph women whose labour was followed by partograph were more than four times more likely to give vaginal birth after previous cesarean section. Correspondingly a study in Northern Ethiopia, Adigrat, reported that labour monitored without partograph was significantly associated with an increased rate of cesarean delivery 24. A study on the effect of the use of partograph on the cesarean section rate has reported that partograph has been associated with a decreased rate of cesarean section 25. This might be because partograph can prevent premature decisions for cesarean section since it shows and follows maternal and fetal conditions with the labour progress.

The study has its own limitations including a possible recall bias while reporting some variables like indications of previous CS and gestational age. Moreover, the result might be affected by the small sample size.

## Conclusion

The study findings indicated that parity, duration of labour, previous history of successful vaginal birth after cesarean section, artificial rupture of membrane, and partograph monitoring of labour were associated with successful vaginal birth after previous cesarean section. Labour should be monitored closely with partograph where possible since partograph can prevent premature decision for cesarean section and the duration of labour should be kept short as much as possible. All Women with a history of cesarean section should be counselled and encouraged to undergo a trial of labour if the indication of the previous cesarean section is recurrent.

## Limitation

The study has its own limitations including a possible recall bias while reporting some variables like indications of previous CS and gestational age. Moreover, the result might be affected by the small sample size.

## List of abbreviations

ANC: Antenatal Care
CS: Cesarean Section
TOLAC: Trial of Labour after Cesarean
VBAC: Vaginal Birth after Cesarean Section
